# The function(s) of consciousness: an evolutionary perspective

**DOI:** 10.3389/fpsyg.2024.1493423

**Published:** 2024-11-26

**Authors:** Thurston Lacalli

**Affiliations:** Biology Department, University of Victoria, Victoria, BC, Canada

**Keywords:** memory theories of consciousness, agency, phenomenal experience, qualia, species memory

## Abstract

The functions of consciousness, viewed from an evolutionary standpoint, can be categorized as being either general or particular. There are two general functions, meaning those that do not depend on the particulars of how consciousness influences behavior or how and why it first evolved: of (1) expanding the behavioral repertoire of the individual through the gradual accumulation of neurocircuitry innovations incorporating consciousness that would not exist without it, and (2) reducing the time scale over which preprogrammed behaviors can be altered, from evolutionary time, across generations, to real-time. But neither answers Velmans’ question, of why consciousness is adaptive in a proximate sense, and hence why it would have evolved, which depends on identifying the particular function it first performed. Memory arguably plays a role here, as a strong case can be made that consciousness first evolved to make motivational control more responsive, though memory, to the past life experiences of the individual. A control mechanism of this kind could, for example, have evolved to consciously inhibit appetitive behaviors, whether consciously instigated or not, that would otherwise expose the individual to harm. There is then the question of whether, for amniote vertebrates, a role in memory formation and access would have led directly to a wider role for consciousness in the way the brain operates, or if some other explanation is required. Velmans’ question might then have two answers, the second having more to do with the advantages of global oversight for the control of behavior, as in a global workspace, or for conferring meaning on sensory experience in a way that non-conscious neural processes cannot. Meaning in this context refers specifically to the way valence is embodied in the genomic instructions for assembling the neurocircuitry responsible for phenomenal contents, so it constitutes an embodied form of species memory, and a way of thinking about the adaptive utility of consciousness that is less concerned with real-time mechanistic events than with information storage on an evolutionary time scale.

## 1 Introduction

Dealing with consciousness from an evolutionary perspective means first addressing the question of how broadly distributed it is across animal taxa, and hence whether, like all other products of evolution, it can be supposed to have evolved progressively over an extended period of time. If so, we can assume that a considerable fraction of extant vertebrate species host some form of consciousness which, if consciousness evolved in parallel with the neocortex or its equivalent, would include mammals, birds and many reptiles (Griffen and Speck, 2004; Cabanac et al., 2009; Allen and Trestman, 2020; Birch et al., 2020; Irwin, 2020; Nieder, 2021; Tomasello, 2022). There is of course the problem of judging the presence or absence of first-person conscious experience from a third-person perspective, but the premise adopted here is that there is no reason that an absence of certainty on this point should preclude an investigation, at least in principle, of how consciousness would have evolved. The subject of this account is the function of consciousness, but the focus is less on the varied tasks performed by our own consciousness today than on what can be deduced about the function or functions of consciousness in the past, in the earliest stages of its evolution. An argument can be made that consciousness has no function (Blackmore, 2016), that it is epiphenomenal, an illusion, or a “ghost in the machine” (Halligan and Oakley, 2021), but this stance is both unhelpful from an investigative standpoint and unlikely from an evolutionary perspective. Rejecting that stance does not, however, resolve the problem raised by Chalmers (1995, see also Morsella, 2005; Rosenthal, 2008), of why the brain might not just as well operate in the dark, i.e., without consciousness. Velmans (2012) deals with this question in an explicitly evolutionary context, so I will refer to it here as Velmans’ question, my premise being that an evolutionary perspective reduces the number of options that need to be considered, and so may simplify the search for an answer.

**Figure 1:**
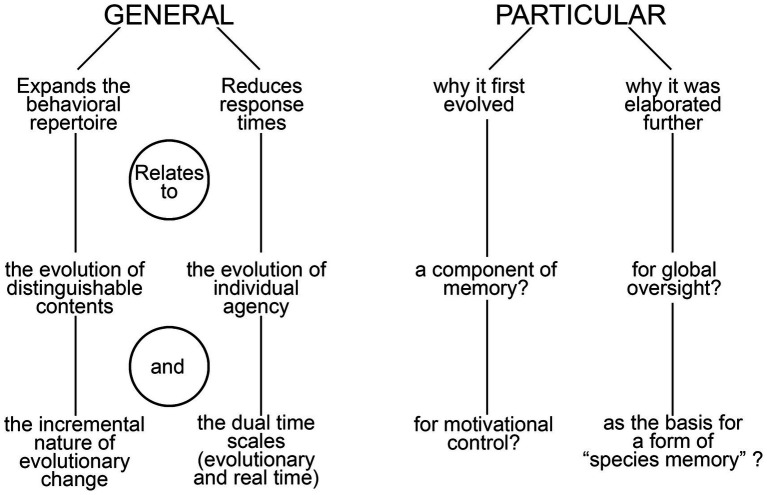
A summary diagram highlighting the main points made in the text regarding the general and particular functions of consciousness. On the side of particulars, a longer list might be expected given the variety of ways our own brain makes use of consciousness, but the core idea is that there is a small subset of these that are the important ones when it comes to explaining the reason consciousness evolved and the advantage it confers over the alternative, of having a brain that operates in the dark. The term “species memory” is introduced to refer to the ability of members of conscious species to recall the conscious sensory experiences of their ancestors, which is possible in so far as the phenomenal qualia involved have remained stable across generations, and hence are re-experienced by each generation as they would have been in the past.

I begin the analysis by distinguishing between two categories of function, general and particular (Figure 1). General in this context refers to the ways consciousness alters behavior irrespective of its utility in a proximate sense, of the specific tasks it first evolved to perform and the sensory modalities and brain functions involved. But there are also particular functions, relating to the latter two points, which I explore more fully in light of a previous analysis (Lacalli, 2024) that highlighted the importance of memory in the conscious modulation of behavior, exemplified by the way negative affect, acting through memory, inhibits appetitive actions in situations where these place the individual at risk. This leads to a consideration of the role consciousness plays in brain function more generally, using the conceptual framework of the global workspace, and from there to a discussion of embodied cognition and meaning viewed from an evolutionary perspective.

The vertebrate skeleton is used at several points in the narrative as a model for thinking about the evolution of complex entities composed of subcomponents that must operate together in a coordinated way, a problem also faced by a consciousness composed of diverse contents. A note also is required on terminology, which can vary depending on authors’ points of view and preferences. The focus of this account is the evolutionary emergence of conscious experience in its simplest form, referred to by some authors as sentience (e.g., Feinberg, 2024), by others as awareness (Ehret and Ramond, 2022), but which in any case is not always easy to define in relation to more fully elaborated forms of conscious experience (Irwin, 2023). I will use “experience” for all of these, including both minimal and more complex contents of consciousness, whether classed as phenomenal, access (Block, 2007), or extended (Damasio, 1999), and regardless of whether they are realized in every stimulus situation. This is because it is the *ability* to have such experiences that is of concern from an evolutionary perspective, rather than how attention to them, or awareness of them, is modulated by other neural processes. The term “consciousness” is used similarly, and in a general way, again to refer to the ability to have conscious experiences regardless of how this manifests itself on a moment-to-moment basis during behavior. What is important also from an evolutionary perspective is having a consciousness characterized by *contents*, plural, meaning distinguishable and more than one. The point here is that we do not know the mechanistic basis of an experiential brain state, but if it resulted from neural processes not too different from those characterizing non-conscious brain function, one or a few fortuitous mutations might have been sufficient to convert a pathway operating in a non-conscious mode into one operating consciously. But the chances of multiple distinguishable contents arising by this means is vanishingly small if the transition from a rudimentary consciousness to one consisting of multiple distinguishable contents can only be accomplished by natural selection acting across multiple generations at a population level, which is almost certainly the case. There must then have been functions for which each of these evolving contents were more adaptive than all available alternatives, which means that there *is* an answer to Velmans’ question, and our task as scientists is to discover that answer.

## 2 The general case

Previous papers in this series identified two functions of consciousness that can be classed as “general” in the sense that they are valid regardless of how or why consciousness first evolved or the brain structures involved: that consciousness (1) increases the range of behaviors possible for the individual (Lacalli, 2021) and (2) reduces by orders of magnitude the time required to change behavior in response to changing circumstance (Lacalli, 2023). On the face of it, both points are so broadly generic that they would be expected in any list of the supposed advantages of consciousness over non-conscious reflexive behaviors, as indeed they often are (e.g., see Cleeremans and Jimémez, 2002; Van Gulick, 2022). And, though they can be combined under the heading of behavioral flexibility, as in the flexible response mechanism of Earl (2014), they are analytically separable, and stand out from other functions attributed to consciousness for being the only ones that arise simply by virtue of the way natural selection acts on brains and behavior across generations.

This requires some further explanation, especially regarding the first point, which is the less straightforward of the two. Range of behavior here refers to all the possible things an animal can do, distinguished also by the mechanistic reasons it does so, since behaviors carried out by different mechanisms are non-identical from a neurophysiologial standpoint even where the outcomes are similar. Since evolution acts at a population level, any increase in behavioral capability due to consciousness will be gradual and cumulative, as evolution explores what is essentially a “cognospace” mapping the behaviors possible with consciousness, in the same way it would explore a morphospace of body form through innovation in skeletal structure. The argument can be formulated more precisely (see Lacalli, 2021) by casting it in terms of the way evolution acts on the neurocircuits responsible for producing a particular phenomenal experience rather than some other, selector circuits in my own terminology (SCs), or difference makers of consciousness (DMCs) in that of Klein et al. (2020). These are a subcategory of neural correlates of consciousness (NCCs), but are content-specific (Mogensen et al., 2020) and causally determinative. The point here, to extend the remarks at the end of the previous section, relates specifically to the divergence of different forms of phenomenal experience, i.e., qualia, and the SCs that determine their individual character: that for consciousness to be adaptive in its influence on behavioral decision making, it must convey information about reality, which it can only do if the qualia of experience are distinguishable in a meaningful way.^1^ This is achieved by their divergence from one or more ancestral ur-qualia along trajectories in a multidimensional SC-space that map the configurations of all possible SCs. Arriving at an endpoint in such a trajectory, and hence the ability to produce and experience a quale with particular adaptive characteristics, can only happen if consciousness is present along the whole trajectory. In other words, that point in SC-space cannot be reached in practice, nor can the corresponding behavioral outcomes exist, unless consciousness itself exists and is not epiphenomenal. In this way consciousness increases the behavioral options available to the individual in ways specific to consciousness that would otherwise not be possible, and regardless of the particulars of what those options happen to be.

The second point is, that so long as consciousness is assumed to confer agency on the individual, that individual can respond more rapidly and flexibly to changing circumstance than it could by depending entirely on non-conscious reflexes. The term “agency” is used here in a quite specific way, not to refer to an agent that is the proximate cause of an action, but to one able to alter a predetermined sequence of reflexive actions to change the outcome, an agent of change in other words, which for the individual equates to volition (Pierson and Trout, 2017). The evolutionary point is that changes to an action sequence can occur in two time scales depending on where agency resides. If it resides with evolution rather than the individual, then such changes happen on an evolutionary time scale, from generation to generation. This would be the case for hard-wired reflexive behaviors, including those that have evolved to incorporate learning and conditioning mechanisms, because the latter only then operate within a predetermined set of parameters. The evolution of consciousness, by transferring agency more fully to the individual, offers an opportunity to move outside that set of parameters, allowing the individual to change the outcome of preprogrammed and non-consciously conditioned behavioral sequences more rapidly than evolution can. The adaptive advantage is then straightforward, of the ability to respond more appropriately and quickly to a real world full of unpredictable events. But again, the reason consciousness confers this advantage, through agency and the mechanisms that make individual agency possible, is both different and separable from the reason it expands the behavioral repertoire, which depends on the evolutionary process by which the distinguishable contents of phenomenal experience are brought into existence.

While both of the above general functions represent positive things one can say about the utility of consciousness, nothing is specified about the neurophysiological mechanisms involved, so an equally adaptive result could in principle be produced by mechanisms other than conscious ones. To answer Velmans’ question we would have to identify a function for which consciousness is more adaptive than all possible non-conscious alternatives, which, given our incomplete knowledge of how brains work at a neurocircuitry level, is at this point in time a daunting if not impossible task. Yet consciousness has evolved in the vertebrate lineage, which is evidence that whatever problem it evolved to solve, it must better than the alternatives in some way. Tackling the problem from an evolutionary point of view then has the advantage that we can focus on the properties of a hypothetical early stage in the evolution of consciousness that can plausibly be supposed to have been simpler than our own consciousness is today. This equates to the minimalist approach adopted by Morsella et al. (2016) and, though a degree of caution is obviously required with suppositions regarding events lodged in the distant past, my premise here is that there are ways of thinking logically about these that reduces the attendant uncertainty. For a brain previously operating in the dark, the question as Velmans frames it, of “what turns the lights on?” gets at the real point of concern, of the events that led to the evolution of an ancestral consciousness of the simplest possible kind. This does, however, highlight the limitations of thinking only in general terms about function, because it is only by understanding precisely how and why consciousness first evolved that Velmans’ question can be definitively answered. Which then moves the argument from the general to the particular, and a consideration of the neural mechanisms and sensory modalities that can plausibly be supposed to have been involved.

A further issue to consider, raised by Polák and Marvan (2019; see also Marvan, 2024), is whether we need also to be thinking about the evolution of non-conscious phenomenal states, where conscious awareness of those states would have evolved only later through a separate set of evolutionary innovations. The arguments introduced above regarding SCs and their evolution would still apply, but to the diversification of non-conscious phenomenal states, allowing access to new domains in behavior space specifically dependent on those states. Why it was advantageous for some of these then to become conscious while others did not would be a separate issue, which means Velmans’ question would have two parts and, possibly, two different answers. This is somewhat different than the issue more often dealt with in relation to conscious versus non-conscious neural processing (e.g., Morsella, 2005; Morsella and Poehlman, 2013), of how these are partitioned in brains already fully engaged in both.

## 3 Particulars: agency, memory, and motivational control

**Figure 2:**
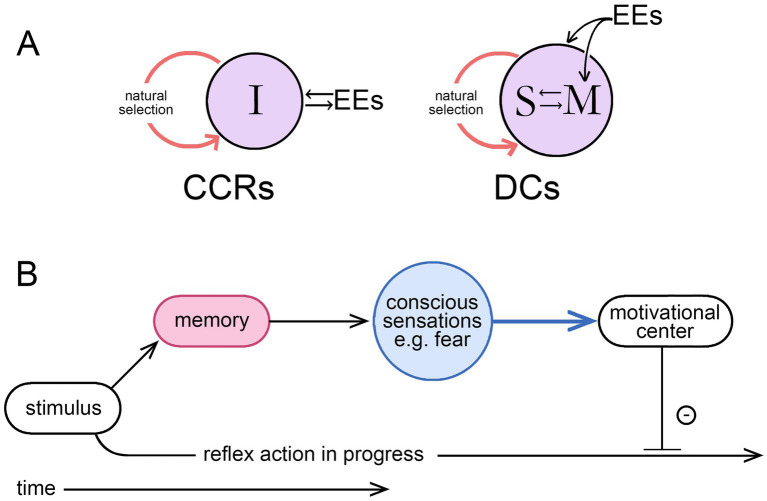
**(A)** The two ways external events (EEs) can impinge on the individual during the real-time learning process required for the evolution of a consciousness endowing the individual with agency, meaning volition in this context; modified from Lacalli (2024), where the argument is more fully developed. Learning through simple conditioning involves the individual (I) in a direct interaction with the external world, but if this involves synaptic plasticity without memory, the process does not confer agency, resulting instead in what are essentially consciously conditioned reflexes (CCRs). Involving memory (M, shown here as interacting with a self-like construct, S) is required for individual agency, where the interaction now occurs in the brain at the speed of neurophysiological processes as this relates to memory access and recall, making deliberate choices (DCs) possible. It is by bringing the interaction into the brain, a step that requires a dedicated memory system, that the limitations of conditioning are overcome, hence the conclusion that any theory of consciousness taking due account of evolution will, at some level, be a memory theory of consciousness. **(B)** A memory-dependent intervention sequence (MDIS) illustrating the point made by Budson et al. (2022) for their memory-based theory of consciousness, that consciously we perceive the world as a memory. Here an olfactory stimulus is used as an example, which, if previously experienced in a situation that generated feelings of fear, will do so again if the sensation and the link to negative affect are lodged in memory. The conscious part of the pathway, i.e., the broadcast function, is shown in blue. On receipt of this, an appropriate adjustment of the motivational control center, to inhibit the action initiated by the stimulus, will then serve to slow or terminate that action. But there is necessarily a time delay as the memory is accessed, so consciousness, in effect, modulates behaviors after the fact rather than initiating them. The idea of conscious “control” over action is then a more subtle proposition than often supposed, which explains the problems encountered in interpreting experimental work on the timing of intentional actions, a topic more fully dealt with by Earl (2014).

This is both a change of topic from the previous section and a logical extension of it. The link is through agency, since it is by means of individual agency that the time scale of intervention is moved from evolutionary time into real-time, a clear advantage to the individual in a world of unpredictable events. But, as above, this means treating agency as referring, not to an agent as the proximate cause of an action, but to the ability to alter a preprogrammed sequence of reflexive actions to change the sequence. For the ability to make such changes to evolve, that ability must be adaptive, but for it to be so, the individual must have a source of information directing it as to how and when to intervene. The result of an intervention would otherwise have no more than a random chance of being beneficial, so a repository of information is implied. If this is not to be entirely preprogrammed into the brain, it must, at least in part, be acquired during the life of the individual. Hence a learning process is required that must occur in real-time. This accords with the idea that consciousness (conscious agency in my formulation) must be learned (Cleeremans, 2011; Cleeremans et al., 2020), or similarly, for theories where consciousness depends on the emergence of a self, that selfhood (agency implied) must be learned and achieved (Marchetti, 2022). The idea is most fully developed in the associative learning model of Ginsburg and Jablonka (2007; see also Bronfman et al., 2016; Jablonka and Ginsburg, 2022), which places associative learning at the core of the process by which motivational states, through consciousness, come under volitional control. One might suppose that this could occur by simple conditioning processes involving synaptic plasticity but not memory, but this, from my previous analysis (Lacalli, 2024), is not sufficient to confer agency on the individual. In contrast, learning processes that incorporate memory storage and recall can (see Figure 2A), a result that accords, again, with the associative learning model. This means, in effect, that for any theory of consciousness to be complete from an evolutionary standpoint it must also, at some level, be a memory theory of consciousness. Judging how well a given theory of consciousness conforms to this requirement then needs to be examined on a case-by-case basis, a task complicated by the presence of two separable time scales, for evolutionary and real-time events, because what is stated as true for one need not be true for the other. This can lead to statements that appear to be incompatible but are not. An example: that consciousness as it evolved may have been dependent on memory, hence the statement above that any comprehensive theory must at some level be a memory theory. But, from a real-time perspective, this does not mean that the neural pathways responsible for producing a conscious sensations in real-time must necessarily access memory in order to do so, which is unlikely in any case (e.g., see Damasio, 1999). The issue here is simply one of being cognizant of the time scale to which a given statement is intended to apply.

An evolutionary formulation also says something important about the encoding of information about the external world through conscious mechanisms: that this occurs in real-time, but also in evolutionary time, the importance of which is in my view underappreciated. The point here is that evolution only has access to reality at one remove, through the effects that reality has on survival and reproductive success. But because of consciousness, information directly related to the hazards of the real world is encoded in the genome in a very specific way, in the assembly instructions for the neural circuits (the SCs) responsible for particular forms of phenomenal experience, i.e., qualia. A character of a given quale, encoded this way, is then an answer to the question “what is the best mediator of a conscious real-time response to a stimulus of a particular kind?” In contrast, memory is the real-time repository of information about reality, but the question is now different: that from all possible behavioral responses, “which, based on past experience, should be chosen from among the available alternatives that evolution has supplied?” There is thus a division of labor between evolutionary and real-time mechanisms, both of which are required for consciousness to be adaptive and useful. The evolution of agency (formally, a link between conscious contents and behavior, see Lacalli, 2023), would then have depended very specifically on innovations at the neurocircuitry level that rendered some component of memory responsive, either directly or indirectly, to the sensations generated by an emerging consciousness.

Consider now the ways memory and consciousness might be integrated in an ancestral consciousness performing the simple function of modulating avoidance behavior in potentially risky situations. Budson et al. (2022) provide a conceptually useful model, an example of which might be as follows. Suppose we have a foraging animal advancing towards a food source in a situation where there are risks from predators. It does not matter for the argument whether the neural mechanism involved in initiating the foraging activity is a conscious one or not, because the conscious component of interest is the one acting to modulate appetitive actions, slowing or redirecting them when current sensory inputs evoke, through memory, conscious sensations indicative of danger. One can think of this as depending on an internal motivational control center, where the motivational state is updated on a continuing basis as new sensory inputs are received. Where this involves conscious sensations, incorporating these into the record of memory, recalling them and applying them to modulate motor pathways takes a period of time, during which the actions initiated by the most recent sensory inputs may already have begun. Appropriate adjustments can be made, but there is always a time lag, which explains the otherwise cryptic statement Budson et al. (2022, p. 270) use to characterize their conception of consciousness, that consciously we “perceive the world as a memory.” This equates to the argument developed at some length by Earl (2014), that choices and decisions are not made consciously, but instead are initiated unconsciously and modulated by conscious inputs as they unfold, or the statement by Wegner (2003, p. 68) that the experience of conscious will is “no more than a rough and ready guide to causation.” Figure 2B illustrates this with a diagram of what I will refer to as a memory-dependent intervention sequence (MDIS), which has features in common with other proposals, notably Damasio’s somatic marker hypothesis (see Bechara et al., 2000). It is the intervention aspect specifically that is important here, that conscious inputs are intervening, with a time delay, to modulate non-conscious processes already in progress. For action sequences that unfold sufficiently slowly, this is adaptive because progress through the sequence can be slowed or arrested in situations where the inhibitory input is sufficiently strong or persists, but will otherwise continue to completion.

**Figure 3:**
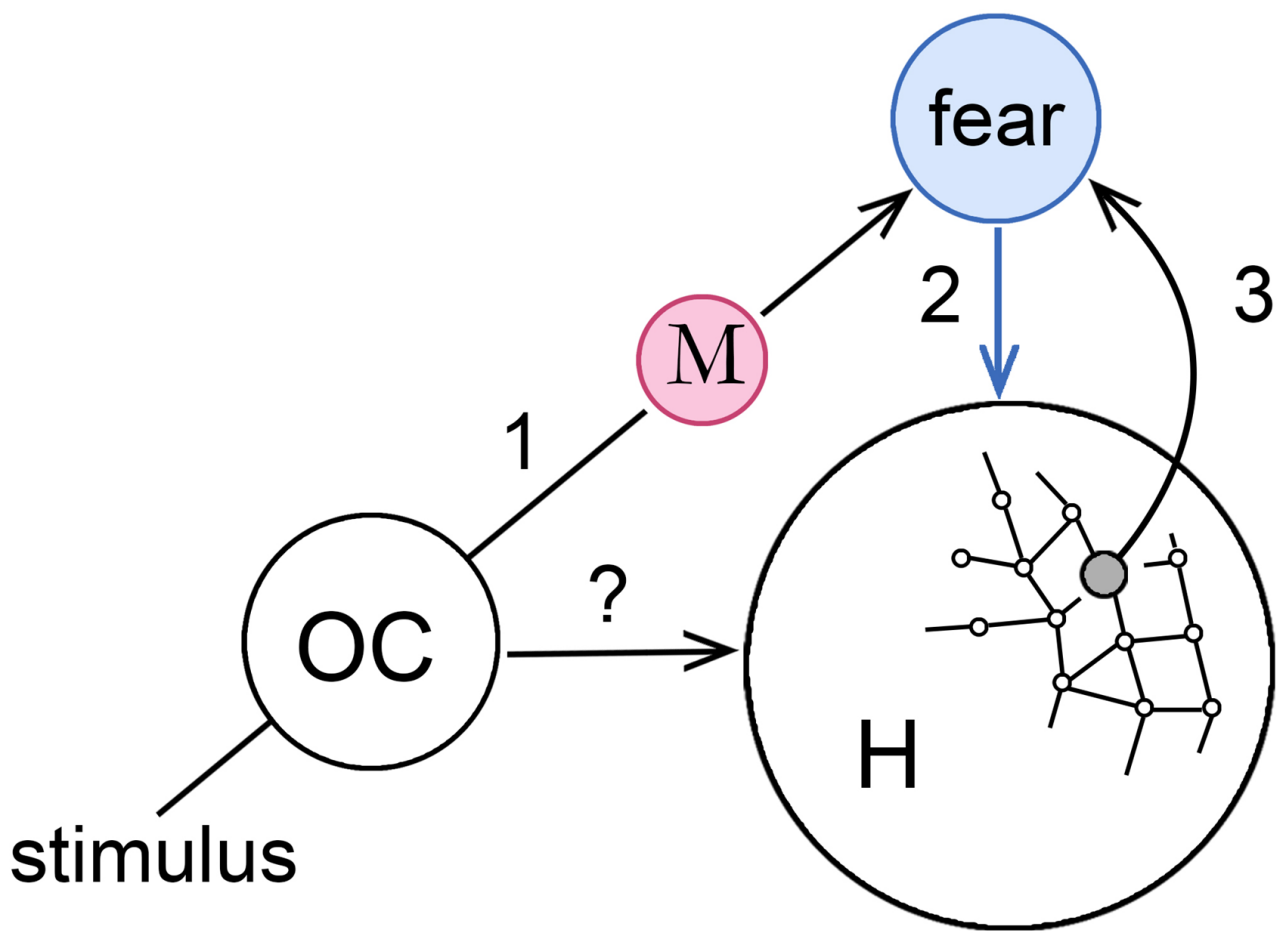
How a memory-dependent intervention sequence (MDIS), modeled on that in the previous figure, might operate in conjunction with place cells in the hippocampus (H), to generate a navigational map of the habitat where each point is assigned an affective valence. The stimulus is, again, an olfactory input to an olfactory center (OC) that could act directly on H (the?), but is linked also via memory (step 1) to a second center, unspecified as to location, that generates an affective sensation (fear in this case, step 2) that acts also on H. The latter effect could be a direct one, meaning H is responsive to the sensation (blue arrow), or indirect via a non-conscious pathway, so the arrow would instead be black. If the olfactory memory is evoked at particular locations in the habitat, activating the relevant place cells simultaneously with the sensation can be supposed to form a link between those place cells and the affective center (step 3) so that the same sensation is automatically evoked whenever the animal occupies that location. This allows the animal in question, perhaps a reptile or early mammal, to use its emerging consciousness to coordinate olfactory experiences, hippocampal navigation, and memory, slowing action at particular places in its habitat, so that more time is available for relevant sensory inputs to accumulate and influence behavior. See Wegner (2003) for a similar diagram relating to his more general discussion of conscious will.

Figure 3 develops the MDIS model further to show how, in principle, it could act to assist an animal in navigating its habitat. The supposition here is that we are again dealing with an emerging early form of consciousness that can be made as simple as one likes, in this case by restricting the argument to a single sensory modality. Any would do, but I have chosen olfaction for the reasons given by Merrick et al. (2014; see also Shepherd, 2007; Baars, 2013; Keller, 2014), that it is at least as ancient as any other sensory modality in phylogenetic terms, but also that the routing of olfactory inputs is simpler than for other modalities in the absence of a thalamic relay. Morsella et al. (2016) also focus on olfaction for the key role it plays in action selection, but my particular interest here is in habitat navigation given the strong linkage between olfactory centers and the hippocampus, hence to memory and, via the orbitofrontal complex, to the amygdala (Jacobs, 2012).

Consider then how a conscious perception of odors might be used to modulate the progress of, say, an early mammal as it forages across its home territory. This would involve activating place cells in the hippocampus as the animal moves, so the operative question is how the MDIS model would apply to an interaction involving cortical centers for processing olfactory inputs and the hippocampus (respectively, OC and H in the figure). Suppose, as the animal moves, it encounters an odor that, based on previous experience now lodged in memory, is associated with a feeling of fear (step 1 in the figure). The co-occurrence of the feeling of fear with a particular location in the habitat, as coded by place cells (step 2), results in a link being formed between the hippocampus and the center responsible for the sensation (step 3) so that, in future, arriving at that point in space will evoke the sensation again. The sensation is shown in the figure as acting directly on the hippocampus, but the effect could just as well be indirect, mediated by a cortical or subcortical center responsive to conscious sensations of affect whose output, via non-conscious pathways, targets the hippocampal complex. A direct effect is somewhat problematic in any case, since scaling it up in an expanded consciousness with more contents would make the hippocampus a central hub for receiving conscious signals, indispensable for consciousness of all kinds, which is not borne out by lesion studies (Squire, 2004: Squire and Wixted, 2011). Regardless of details, as a result of repeated events of the kind illustrated in the figure, the brain’s internal navigational map would acquire an overlay of affective content that informs the individual of the valence associated with each point in space. Using this, foraging activity could then be redirected along paths of least risk. As a model for how an early form of consciousness might have operated, this combines, as required, learning and memory, but Velmans’ question remains unanswered for the same reason as in the previous section on general functions: that given the variety of ways neurocircuits can be configured and the functions these could conceivably perform, there could be non-conscious mechanisms that are just as effective as conscious ones for memory-based motivational control.

Something of a leap of faith is then required to suppose that consciousness has an advantage, so I will make a lesser claim: that of the various ways that motivational control might operate, relying on a combination of consciousness and memory is one, and this is adaptive for at least some subset of functions performed by the brain that involve memory encoding and recall. Two reasons why then suggest themselves, that (1) consciousness aids in tagging memories for later recall, essentially as part of a filing system that operates more effectively when the tags are conscious rather than not, or, to focus on the recall process, that (2) bringing the memory or some form of it into consciousness makes that memory more effective as a modulator of motivational state than it otherwise would be. I have no argument to advance to choose between these options, but there is considerable empirical evidence that consciousness plays a role in both (La Bar and Cabeza, 2006; Buchanan, 2007), so it may be through investigations of how memories are encoded and recalled that the issue will be resolved. This should include a consideration of prediction models of consciousness (den Ouden et al., 2012; Keller and Mrsic-Flogel, 2018) where conscious awareness of prediction errors serves to highlight those errors, and where the expectation against which the error is measured is a product of learning and memory. The higher-order nuclei of the thalamus appear to play a central role here, especially in relation to the conscious visual display (La Terra et al., 2022; Whyte et al., 2024), a complication being that the visual display, due to its internal structure, is analytically in a separate category from phenomenal contents (it is a format in my terminology, see Lacalli, 2021), so deductions based on the properties of conscious vision would not necessarily apply to simpler conscious contents.

## 4 Extending the memory argument

If we begin with a limited form of consciousness adapted for the memory functions described above, and given that learning and memory operate both with and without consciousness (Cleeremans and Jimémez, 2002; Squire and Dede, 2015), the next question is what accounts for the wider role that consciousness has come to play in decision-making in brains like ours. To evolve as simply one of many brain mechanisms, we require only that a conscious response was better than a non-conscious one at some point in time in some particular circumstance whereas, to be elaborated further as a pervasive mode of brain function, the advantage must be one relevant to the way the brain is organized and functions more generally. Repeated tests by evolution have confirmed that there is such an advantage, as our brains would otherwise not employ conscious pathways as widely as they do. But the point here is that all evolutionary innovations are available to be coopted for purposes other than the ones for which they first evolved, so there may again be two answers to Velmans’ question as it relates to particular functions (see Figure 1): of (1) why consciousness evolved in the first instance, and (2) having done so, why it was elaborated further. Assuming the answer to the first question relates in some way to memory storage and recall, what explains the second? I will suggest two possibilities, one derived from global workspace theory (GWT, see Baars et al., 2013; Baars and Alonzi, 2018), here combined with its neuronal workspace variant (Mashour et al., 2020), the other from a consideration of information storage on an evolutionary time scale.

Of the various theories devised to account for consciousness (see Seth and Bayne, 2022), few deal thoroughly enough with evolutionary issues to fit easily within the framework developed here. Integrated information theory (ITT) is the poster child in this respect, a theory I would class with computational theories of consciousness in having minimal connection to neurobiological reality. Whether ITT can be considered a theory of consciousness at all is addressed elsewhere (Merker et al., 2021), but it is possible that a memory-based theory of consciousness could, at a foundational level, depend on something resembling IIT, or for that matter a representational theory, or one of the various EM field-based proposals for explaining consciousness. But these aside, there is one theory that stands out for its applicability in the present context, and that is GWT for the explicit connection it makes between working memory and consciousness (Baars and Franklin, 2003; McFadden, 2023), a feature that also has considerable empirical support (Morsella and Poehlman, 2013). It is then an easy step from an ancestral motivational control system dependent on memory access and recall, as outlined in the previous section, to something resembling a global workspace, with more inputs, but operating through similar mechanisms. Because GWT is chiefly concerned with the way consciousness is organized and functions, its success (or failure, see Block, 2009) in dealing with the hard problem and related foundational issues is not a concern. Only operational issues are relevant, among which is the dependence of the global workspace on a broadcast mechanism by which sensory information, once in the workspace, is available to be assessed and acted on by more specialized cognitive processes (Baars et al., 2013). Conscious awareness would then be either directly involved in the interactive process, or emerge in consequence of that process, depending on interpretation (Morsella et al., 2016; Raffone and Barendregt, 2020). Since the focus of this account is on function rather than operational issues, the term I will use here for this kind of function is global oversight, which could either be a self-organizing process, as in most formulations of GWT, or require a self-like entity (e.g., see Marchetti, 2022) to act as an overseer. Unresolved issues with GWT include the role phenomenal contents play in the workspace (Raffone and Barendregt, 2020) and, if one chooses to think in terms of access, as in access consciousness (Block, 2007), whether access is to actual contents or to some other kind of mental construct (Kemmerer, 2015). Rather than enter this debate, my preference here is to deal with global oversight and the workspace concept in general terms, making it potentially applicable to all conscious contents including phenomenal ones.

What then are the advantages of arranging for a global oversight function to be carried out consciously rather than not, or, as Morsella et al. (2016, p. 5) pose the question: “what is the most basic form of integration that requires consciousness?” A first point is that hierarchical organization, where some levels exercise oversight over others, is a widespread feature of neurobiological control systems, and is assumed to allow such systems to operate more flexibly when it comes to problem-solving or choosing an action sequence (Badre and Nee, 2018; Macpherson et al., 2021), especially where the subcomponents of the process operate on different computational levels (Kashtan and Alon, 2005; Meunier et al., 2010). The question to ask of GWT is why the top level in the neural hierarchy, the one responsible for global oversight, should be conscious while lower levels of the hierarchy are not. The most convincing answer to my mind comes from the analysis by Mengistu et al. (2016), which clarifies the reason hierarchies evolve in the first place, but also why there could be an advantage to having the top level operate in a mechanistically distinct way. The argument is based on connection cost, that hierarchical organization minimizes the cost of establishing and maintaining hard-wired synaptic connections in comparison with a system that is not hierarchically organized. If consciousness, acting in a broadcast mode, can then replace yet more of the synaptic connectome, there should be an even greater cost savings. Hence the relevance of the broadcast metaphor for GWT, that the cost advantage should be comparable to that of a radio transmission over a fixed line phone network, or Wi-Fi over a dedicated cable connection. Not knowing how conscious signals are actually received and translated by neurons into action, we have no way of estimating the actual costs involved, or the range over which the broadcast operates, but for a connectomal model of consciousness all that is needed is for the broadcast function to be more cost effective than the hard-wired alternative.

Regardless of theoretical stance, a cost-based analysis of any theory of consciousness built around a broadcast model must deal with two separable functions, of producing a signal and responding to it.^2^ In the GWT framework, the signal equates to information entering the workspace, being “posted” so to speak, while the response is the process of accessing and interpreting that posting and doing something about it. Assuming consciousness does confer a cost savings, and regardless of theoretical stance, the further point is that the greatest saving should be at the level where greatest range and flexibility are required, i.e., at the top of the control hierarchy, and only there. This is because, if consciousness operates too widely across other levels of the hierarchy, and assuming a limit to the number of contents a consciousness can accommodate without interference, the differential advantage, of reducing cost where it is greatest, would be lost. There is also the question of whether one way of reducing the costs associated with synaptic connections might be to have the broadcast function depend on extra-connectomal mechanisms, such as EM-field effects. The relevant point here from a control standpoint is the difference between synaptic inputs, where it matters whether the synapse is excitatory or inhibitory, and EM field effects, whose signal strength scales with current density regardless of whether those currents arise from excitatory or inhibitory neurons (Hales and Ericson, 2022). Given these two options, it would be surprising if there were not situations where this difference between them proved advantageous as a way of decoupling connectomal and extra-connectomal modes of control. A problem with an extra-connectomal form of broadcast is, however, that the cost advantage is lost if conscious and non-conscious EM field effects employ the same mechanism, so the advantage of one over the other would require another explanation.

A second, related argument is that consciousness provides a common currency for information exchange (equally, a *lingua franca*, see Morsella et al., 2016), and hence a way of quickly summing the contribution of multiple inputs from different sources and sensory modalities. Cabanac (1992, 1996) has argued that pleasure plays this role, and Cleeremans and Tallon-Baudry (2022) for a property they refer to as value. Having valence play this role for a global oversight function is then a kind of accountancy, of keeping score in a way that does not interfere with all the other functions the underlying circuitry is required to carry out. This problem, essentially the cost of interference, deserves some further comment. Suppose we have a neural circuit dedicated to solving a set of specific computational problems unconsciously, where the structure of the circuitry is selected to optimize that function and no others. If optimization means maximizing the speed at which those dedicated computations are performed, there is a risk that incorporating a conscious component will slow the computation if the neurons involved are also required to generate and/or respond to the assortment of synchronized waves and spiking patterns on which consciousness is supposed to depend (Varela et al., 2001; Sauseng and Klimesch, 2008; Hunt and Schooler, 2019). The advantages of consciousness in this circumstance, whatever those are, would then have to outweigh the disadvantages of reduced computational efficiency.

The discussion to this point has focused primarily on operational issues, specifically how global oversight could be carried out most efficiently. There is, however, an alternative way of thinking about the function of consciousness that relates, not to mechanistic considerations, but to meaning. This is indicated on the lower right-hand-side of the diagram in Figure 1, as providing a reason why consciousness was expanded and elaborated following its first emergence, but it could equally well explain, in Velmans’ terms, that first emergence. “Meaning” in this context refers to the way encoding a memory so as to incorporate information on valence can be said to embody meaning about the real-life experience that generated the memory. This is the basic premise of embodied cognition theory (Johnson, 2017; Shapiro and Spaulding, 2021) and related ways of grounding cognition (Barsalou, 2020). The embodied cognition counterpart to the statement by Cleeremans and Tallon-Baudry (2022), that phenomenal consciousness has intrinsic value, would then be that phenomenal consciousness embodies meaning. There are two issues to consider here, what we mean by information, and what it means for information to be embodied. As to the first, consider that valence exists for affective phenomenal contents only because of the evolution of distinct qualia, some of which will be positive and others negative. These have evolved as they have to enable adaptive responses to real life situations, in the simplest case by modulating between approach and avoidance behavior. So there is information here regarding situations that are hazardous versus those that promise a reward.^3^ In real-time this information is encoded for the individual in memory, so that, for example, the sudden appearance in the visual field of a large moving shape with stripes and sharp teeth is endowed, via the quale of affect this experience evokes, with a specific meaning: that it is best avoided. This then begs a second question, of how something non-material like a sensation can be “embodied” if this is taken to mean that it has a material counterpart. But in fact it does since, for a brain operating in real-time, there would be no valence without the neural circuitry that selects a particular quale over all others, i.e., the SCs referred to in previous sections. But evolution has access to information here as well, encoded in a different way, in the genomic instructions required to assemble those SCs. Meaning is then, in effect, encoded in two places and two time scales, in memories of past experience in real-time and in the instructions for assembling SCs in evolutionary time, where these two encodings are co-dependent. The key point, to ground the argument in evolutionary terms, and in evolutionary time, is that a new category of assembly instructions for brain neurocircuitry has been brought into being by the evolution of consciousness. These are unique among all other contents of the genome in being a genomic embodiment of meaning, the cognitive component of the genome so to speak, and it may be this, as an innovation, that makes consciousness adaptive.

The argument in the preceding paragraph shifts the focus from a concern with mechanisms to information storage, of where the information in question is stored and the form it takes. For the encoding of qualia-related information in the genome I suggest the term “species memory” because the information in this instance derives from the accumulated past experience of members of the species in question, across many generations, that is then placed at the disposal of each individual, first as its brain develops postnatally, and after that on a continuing basis. Each individual can then “recall” its ancestors’ past phenomenal experiences by re-experiencing them. This is part of the heritage of the species in the same way that employing calcium in the construction of the skeleton would, for a vertebrate, be part of its heritage, the difference being that species memory is the experiential part of that heritage. Velmans’ question would then be answered if one accepts that having more information is in practice better than having less. As an aside, it follows that the deficit for machine intelligence is the inability to assign meaning to inputs without specific instructions as to how to do so. For a conscious biological species, meaning is assigned as consciousness evolves, so in effect, the species instructs itself over an extended series of generations in a way that machine intelligences are not as yet designed to do.

## 5 A digression on the uses of theory for investigating animal consciousness

The practice of science combines two complementary activities, of making observations and devising ideas to explain those observations or, in more formal terms, experiment and theory. The study of consciousness, though arguably not yet a fully scientific enterprise, nevertheless supports an abundance of theories, the problem being that there is as yet no generally accepted way of choosing among them. There is also a wealth of experimental work, notably directed at the neural correlates of consciousness (NCCs) with the goal of determining what role these play in integrative pathways underlying conscious experience (Nani et al., 2019; Friedman et al., 2023), ideally to apply these to investigate species, unlike ours, incapable of verbal report (Ehret and Ramond, 2022). A difficulty with this approach is that we do not know how NCCs map to neural processes operating in a conscious mode, if in fact they do. The integration consensus (Morsella et al., 2016) takes the positive view, that they do, in accord with the widely accepted view that “consciousness is ‘big’” in the words of Blumenfeld (2023) in depending on interconnected cortical networks operating at scale (Bressler, 2008; Petersen and Sporns, 2015; Ito et al., 2022), linked by coordinated patterns of activity, waves and resonance effects of various frequencies, phases and strengths (Varela et al., 2001; Sauseng and Klimesch, 2008). But despite the evident importance of the cortex for sensory processing and memory (Merker, 2004) there is no proof that the neurocircuitry responsible for generating conscious experiences co-localizes with any of these patterns of activity, so consciousness itself could reside elsewhere (Merker, 2007; Morsella et al., 2016, footnote 2). Cortical NCCs would then be of limited use when it comes to explaining consciousness as a phenomenon or how consciousness first evolved, which may be the case for multiple categories of NCCs in any case (Neisser, 2012; Hohwy and Bayne, 2015; Overgaard and Kirkeby-Hinrup, 2021). The whole issue is especially problematic for those taking a different view of consciousness, that it is not big, but small, meaning constructed of smaller modules, or micro-consciousnesses (Zeki, 2003), each independently able produce conscious sensations. Explaining how any one of these modules functions, regardless of its location, would then be sufficient to explain consciousness as a phenomenon.

Modularity and localization are thus central issues that get to the heart of the evolutionary questions that need answers, as to the minimal neural circuitry required for any form of consciousness to exist and, for a newly evolved consciousness, where those circuits would have been located. For a modular consciousness, incorporating new contents would simply be a matter of replicating the basic module, as it would for any consciousness-related subprocesses, including the self for theories that require one. The option for all such functions is that they are either non-local and indivisible, or local and replicable, which then has practical consequences for those investigating neural architecture in high-resolution reconstructions (e.g., Shapson-Coe et al., 2024) if this means that insights into the structural and neurophysiological basis of consciousness can be obtained by investigating considerably less than a whole brain, and assuming one knows where to look. But until the issue of localization is resolved, both theory and experiment face the problem of not knowing if their explanatory targets are the correct ones.

For the frustrated theoretician there is, however, an alternative way forward in the form of the thought experiment, a logical exercise that does not depend on a commitment to any one theoretical framework, but simply follows a line of argument to its logical conclusion. Einstein’s original paper on special relativity is a well-known example, illustrating the logical consequences of accepting the premise that light travels at a fixed velocity irrespective of inertial reference frame. The arguments I use in this paper, especially in section 2 on the general functions of consciousness, are presented in the same spirit, and I stress this point to justify the occasional complexity of the exposition, with its caveats and qualifying remarks, because that is the nature of the exercise. The core of the argument in section 2 depends on two suppositions, which I will call “facts” for being as close to facts as one can expect for a subject as nebulous as consciousness. Each then leads to a general function. Fact one is that biological consciousness is a product of evolution, a process whose main features are an accepted part of current scientific understanding. Among these is that complex structures do not emerge suddenly by single mutations but by the gradual accumulation of genomic changes at the population level. Brains are no exception, nor are neurocircuits (the SCs or DMCs) that are causally responsible for selecting one phenomenal sensation or brain state over another. Hence the existence of distinguishable phenomenal contents is a crucial indication both of the action of evolution across generations, but also that we are dealing here with real causal effects that can be subjected to further investigation.

My second point, and the second fact, relates to time scales, that by introducing into reality an iterative cycle of birth, reproduction and death, evolution has also introduced a new time scale, of evolutionary time, measured from generation to generation, that is insulated to a degree from real-time events. It is in this context that agency is best understood, that one function of consciousness is to transfer agency from evolutionary time to real time, and hence to the individual, which then has knock-on effects in terms of expanding the role learning and memory can play in behavioral control. But here again it is not the general function that is relevant to answering Velmans’ question, but the particulars, of what made this evolutionary step both possible and more adaptive than all available alternatives. Further, there could be two such answers if, as pointed out in previous sections, consciousness first evolved for one function but was secondarily coopted to perform a second. This possibility, of a two-step sequence, has a parallel in the experimental data on cortical NCCs, which also implies a two-step sequence. Ehret and Ramond (2022) summarize the case: that conscious perception of a stimulus begins with awareness, or the potential for awareness, and then proceeds, as a second step, to render the perception of the stimulus fully conscious. The second step is associated with a measurable time delay and is represented by a different pattern of NCCs, which the authors interpret as evidence for an evolutionary sequence: of the emergence first of the *ability* to host conscious perceptions (*awareness* in their terminology), followed by the incorporation of this ability into pathways operating on a more global scale, but with a gatekeeper function that controls, through selective attention, the contents of each fully realized conscious state. This would appear to map to the distinction drawn in my analysis, between an initial function for phenomenal experience in memory-dependent motivational control, and a later, expanded role in global oversight, a congruence between experiment and theory that may be more than a coincidence.

## 6 Conclusion

A central concern in understanding the putative function or functions of consciousness is Velmans’ question of why the brain operates in a conscious mode at all, rather than in the dark, or more specifically, in evolutionary terms, of what first turned the lights on. It is sufficient then to account for sentience of any kind, a minimal awareness of experience, and ask what function that earliest form of consciousness performed. Two categories of function are described here, general and particular, but the former category is so broad as to include almost any advantage consciousness might be supposed to confer. What is required instead is a way of tying the first emergence of consciousness, perhaps of a minimal kind, to a particular adaptive challenge where having neural pathways operating in a conscious mode proved better than the alternatives. With respect to particular functions, based on this analysis and the one preceding it (Lacalli, 2024), the best case in my view is that consciousness evolved first as a device for incorporating learning into behavior in a novel way, allowing better choices to be made between alternative actions than was possible using non-conscious pathways, whether those involved learning or not. This necessarily leads to a consideration of memory, because simple conditioning without memory is not enough to produce consciousness with agency. In consequence, for a theory of biological consciousness to be consistent with evolutionary process, there would appear to be no alternative to its being, at some level, a memory theory of consciousness. But consciousness has also acquired an expanded role in the global oversight of behavior, which begs the question whether there are other advantages of having brain circuits that operate in a conscious mode that are specific to this oversight function. Two potential mechanistic advantages are discussed here, of minimizing connection and interference costs, but an equally strong case can be made in my view for the ability to assign meaning to sensory stimuli, an option that more fully accommodates memory as core component. I cannot prove this, but my analysis does lead to a way of recasting the issue to show how central it is to any discussion of consciousness in an evolutionary context. To make the point as clear as possible, I will state it as a conjecture and then defend that conjecture: that of the particular functions one might suppose that consciousness performs, a key one, related both to memory and to the existence of distinguishable qualia, is to assign meaning to sensory inputs. The supporting argument is as follows: that as individuals we have real-time sensory experiences that are encoded in memory in one form or another. But as a species, we also have “species memory” whereby the experiences of past generations are encoded in the genome in the form of assembly instructions for the brain circuits responsible for evoking particular sensations (the selector circuits, SCs, or DMCs in the terminology of Klein et al., 2020) that define the characteristics of phenomenal experience, i.e., qualia. Species memory is, in effect, a way for the genome to record information on what events in the real world are “like” in the sense that for each, there is a best way to respond, and the sensations that have evolved are those that evoke the most suitable response in each case. Each quale, by having specific characteristics, is then a source of information about the real world that is part of the developmental toolkit made available to each individual, benefitting that individual as it confronts the contingencies of life in the real world. There may be alternative ways of achieving this without consciousness, the key question being whether any of these confer meaning on sensory inputs in the same way that species memory does.

Approaching the problem this way then says something quite specific about “meaning” in an evolutionary context: that it derives from what the individuals of the species, collectively, have learned about reality, validated through repeated tests where survival and reproductive success are at stake, encoding the results on an evolutionary time scale in species memory. The same would apply to more complex contents as those evolved, that genetic instructions required to generate the neural structures that produce those more complex contents are further additions to the cognitive content of the genome, and hence to species memory. Whether this provides an adequate and sufficient answer to Velmans’ question is at this point unresolved, so other options cannot be ruled out. But to explain both why consciousness first evolved and why it was subsequently elaborated further, as it has been in brains such as ours, the benefit of endowing experience with meaning is in my view a serious contender.

## Data Availability

The original contributions presented in the study are included in the article/supplementary material, further inquiries can be directed to the corresponding author/s.

## Footnotes

^1^This account focusses on phenomenal contents to the exclusion of more complex contents, in part because of the assumption that they evolved first, but also because the argument made here cannot be extended in a straightforward way to include complex contents. For the reason, see Lacalli (2021).

^2^Since all broadcast models, including GWT, are basically signal/response models, the terms can be used interchangeably, as I do here.

^3^See Earl (2014) for an extended discussion of qualia as a form of information, and Marchetti (2018) for the unique nature of the information consciousness makes possible.
